# Hit or Miss?: Benefits and Risks of Using Nanoparticles for *in Situ* Remediation

**DOI:** 10.1289/ehp.117-a552a

**Published:** 2009-12

**Authors:** Adrian Burton

**Affiliations:** **Adrian Burton** is a biologist living in Spain who also writes regularly for *The Lancet Oncology*, *The Lancet Neurology*, and *Frontiers in Ecology and the Environment.*

Nanotechnology holds the promise of vastly expanding our ability to clean up hazardous waste sites and decontaminate polluted ground-water *in situ*. Polychlorinated biphenyls, organic solvents, petroleum products, arsenic, and many more contaminants are on the list that specifically engineered nanoparticles could rapidly remove from contaminated soil and water, saving billions of dollars that would have been spent on more expensive conventional remediation methods. These possibilitites are discussed in a review of field tests using nano-materials **[*EHP*** **117:1823–1831; Karn et al.]**. However, the authors caution, our knowledge of the potential environmental and health hazards posed by these nanomaterials is in its infancy.

In the United States alone there are hundreds of thousands of sites contaminated with hazardous wastes, with more than 1,200 requiring priority attention. Using traditional remediation technologies, such as pumping out and treating contaminated groundwater and removing contaminated soil, the job of cleaning up U.S. hazardous waste sites could take 35 years and $250 billion. The authors of this review, however, report on results showing that nanoscale zero-valent iron (nZVI) nanoparticles could dramatically reduce the time required to remediate soil and water as well as, according to one report, save 80–90% compared with conventional methods.

Although tiny in diameter, the surface area of these iron-based nanoparticles reaches 20–40 m^2^/g. This relatively large surface area can greatly increase the particles’ reactivity. Flowing with the groundwater they can spread out to react with pollutants, transforming them into safer compounds, including compounds that bacteria can break down. The authors provide many examples of how nZVIs, currently the nanomaterials most widely used for *in situ* remediation, produced measurable effects within days or, in some cases, hours.

However, the authors also emphasize that we do not know much about the potential adverse effects of nano-particles deployed into the environment—agents that could, for example, end up in our drinking water. Some nanomaterials have already been found to enter organisms. Might some be toxic or transport bound pollutants to places they might not otherwise have gone? Can they be biomagnified? How do they affect living organisms?

The authors point out that, although the environment is full of naturally occurring nanoparticles, manufactured nanoparticles may behave in unpredictable ways. They recommend that while we improve engineering applications using nanoparticles for *in situ* remediation, we also develop the analytical tools to enable the study of manufactured nanoparticles in the environment and increase research on the ecosystem effects of these materials.

## Figures and Tables

**Figure:**
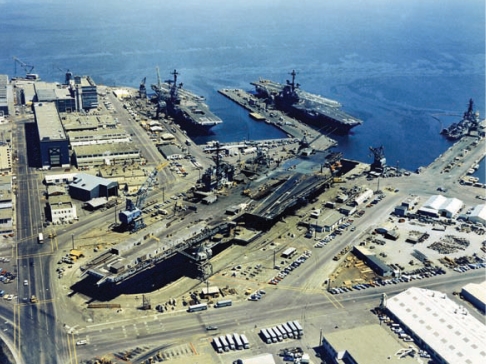
A comparison of remediation using nano- versus microscale zero-valent iron particles at Hunters Point Naval Shipyard (shown here in 1971) showed that each size had unique advantages.

